# A seven-million-year hornblende mineral record from the central Chinese Loess Plateau

**DOI:** 10.1038/s41598-017-02400-0

**Published:** 2017-05-24

**Authors:** Tong He, Lianwen Liu, Yang Chen, Xuefen Sheng, Junfeng Ji

**Affiliations:** Key Laboratory of Surficial Geochemistry, Ministry of Education, School of Earth Sciences and Engineering, Nanjing University, Nanjing, 210026 China

## Abstract

Previous studies of the late Cenozoic erosion rate have yielded different views—long-term stable rates or a significant increase at climate transitions—leading to uncertainty concerning the hypothesized global erosion rate controlled by either tectonic uplift or climatic changes. Here, we present a seven-million-year hornblende mineral record along the Lingtai section of the Chinese Loess Plateau. By examining the spatial distribution of hornblende minerals in seven desert basins, which are potential loess source areas, we constructed a ratio of hornblende versus total heavy minerals to reflect past changes in physical/chemical weathering strength. Our results demonstrate that the ratio has generally increased since 7 Ma, with three significant shifts recorded at 2.6 Ma, 1.4 Ma and 0.5 Ma linked to the onset, continuation and expansion of the Northern Hemisphere glaciation, respectively. Given that chemical weathering during the diagenetic history produces a trend of smoothly increasing hornblende migrating upwards, the three shifts at these boundaries can be interpreted as changes in the bedrock erosion rate on the northern Tibetan Plateau, which may be related to tectonic uplift events and incision of the Yellow River. Evidence presented here supports the idea of coupling between climate change, tectonic uplift and regional erosion.

## Introduction

Erosion of mountains by rivers, glaciers, and landslides constantly counteracts plate tectonic-driven mountain building processes; these erosional processes also contribute a significant amount of atmospheric CO_2_ drawdown to the global climate through the weathering of silicate minerals^1^. Tectonic uplift during the late Cenozoic Era created steep mountain slopes and increased erosion rates^2^, which may have caused the significant increase in oceanic sediment accumulation during the past few million years. A strong cooling in the Earth’s climate 2–4 million years ago is believed to be linked to the development of Northern Hemisphere ice sheets and mountain glaciers at high latitudes, which may have triggered an increase in global erosion^3^. This view is supported by evidence of a widespread increase in the erosion rates of mountain belts globally at the onset of the Northern Hemisphere glaciation (NHG)^4^.

Linking the individual contributions of tectonic and climatic factors to erosion rates has been a long-debated issue^5, 6^. Results from exposure dating of cave sediments^7^ and low-temperature thermochronology^8^ in the European Alps suggest that erosion increased abruptly at approximately 1 Ma, close to the mid-Pleistocene Transition (MPT). Modelled data support a link between climate and erosion by revealing significant increases in glacial erosion in the Alps across the MPT due to feedback between mountain topography and glaciers^9^. Spatially distributed thermochronometric data also provide evidence for an increased global erosion rate coinciding with the onset of continental glaciation in the Northern Hemisphere^4^. Studies in both New Zealand and British Columbia, Canada, reveal a significant increase in mountain erosion at 1.8 Ma^10, 11^ linked to a global transition of increased glacial–interglacial climate variability^12^. To date, few studies have revealed a complete and continuous history of increased erosion and weathering in mountain belts that is coupled to potential triggering factors, such as climate changes, regional tectonic uplift and geomorphological changes, during the late Cenozoic. In contrast, oceanic ^10^Be/^9^Be isotopic data records suggest long-term stability in global erosion rates during the late Cenozoic cooling^13^, resulting in questions as to whether an increase in global erosion actually occurred at this time.

The Chinese Loess Plateau (CLP) contains the largest accumulation of loess in the world. This highly homogenous loess originated from denudation of large mountain areas and represents the average composition of upper continental crust^14, 15^. Systematic investigations of Nd–Sr isotopic signatures indicate a loess source region located in the high mountains around the northern Tibetan Plateau (e.g., the Qilian Mountains and Kunlun Mountains). Tectonic evidence suggests that these young orogenic belts have exceeded an altitude of 4000 m since the late Pliocene^16^, a height well above the equilibrium-line altitude for snow/ice. During periods of Eurasian ice sheet advance, mountain glaciers developed in these loess source regions, resulting in the enhancement of glacial erosion and deposition of a large amount of silt in the lower desert basins. These fine-grained particles were thoroughly mixed by long-distance wind transport to the CLP. Palaeomagnetic stratigraphy^17^ indicates that the thick aeolian deposits on the CLP extend back to the Late Miocene (approximately 7 million years ago) and primarily consist of Pliocene red clay and Quaternary loess sequences. These thick loess sections provide a rare opportunity to investigate the long-term history of bedrock erosion from mountains.

## Materials and Methods

In this study, we investigated the sequence of aeolian dust deposits at Lingtai (35°04′N, 107°39′E) in the central Loess Plateau (Supplementary Fig. S1). The Lingtai section is 650 km to the southeast of the middle Qilian Mountains. Considerable work has been conducted on palaeo-monsoon changes^17–22^ and dust provenance shifts^23, 24^ at the Lingtai section and the nearby Chaona section. The chronology of this section has been determined using palaeomagnetic reversal sequences^17^ and was further characterized by magnetic susceptibility and grain size data^18^. Seventy-two samples from the Lingtai loess–palaeosol sequence, spanning the past seven million years, were selected for Mineral Liberation Analysis (MLA) (Fig. S2). Fifty-seven samples were analysed from the Quaternary loess deposits to achieve a sufficiently high sampling density to resolve glacial–interglacial variations. Fifteen samples from the underlying Late Miocene–Pliocene red clay sequence were also measured to broadly characterize the sequence.

Seven deserts, including six Chinese deserts and the Mongolian Gobi Desert, were also investigated as potential source areas for the loess deposits (Fig. S1). We collected a total of 30 surface sand samples (within the upper 20 cm) to determine the spatial distributions of the sandy minerals.

Using chemical pretreatment and the MLA technique (Methods Summary, and Fig. S3), we measured the abundances of all minerals in the 28–75 µm grain size fraction. A total of 40,000 grains were analysed from each sample to achieve high precision in mineral content with uncertainties as low as 0.01%.

## Results

A total of 23 mineral species were identified in each sample. Among the minerals identified, quartz and plagioclase (presented here as the sum of all plagioclase sub-species) are the most abundant, accounting for 50.2–72.1% and 13.6–24.5% of the samples, respectively. In the secondary group of mineral species, nine heavy mineral species comprised 1.85–4.74% of the mineral content (Supplementary Material, Dataset 1D). Over 90% of the heavy mineral fraction was composed of hornblende, epidote, rutile and titanite. The dominance of these species in the heavy mineral assemblage of the loess deposits is supported by previous studies of heavy minerals from multiple sites on the Loess Plateau^25^. This assemblage generally originated from intermediate–acid magmatic intrusions. This lithology is widely distributed in the loess source regions; furthermore, it has a larger proportion in the Mongolian Altay Mountains than the mountains along the northern Tibetan Plateau^26^.

Hornblende commonly occurs in crystalline rocks and is likely to be weathered under Earth’s surface conditions^27^; therefore, variations in the hornblende content of the loess reflect changes in either physical erosion of the crystalline bedrock in the loess source area and/or chemical weathering of the loess deposits. The hornblende content along the Lingtai section generally increases over time (Supplementary Fig. 4S-a). This long-term trend in mineralogical abundance agrees well with changes in the ratio of plagioclase to quartz and the percentage of anorthite in the total plagioclase species composition along the same profile (Supplementary Fig. 4S-a).

Here, we use the ratio of hornblende versus total heavy minerals (hereafter denoted as R_h_) to represent changes in the strength of physical weathering in the loess source region (see discussion below) and/or chemical weathering during loess diagenesis. In contrast to the changing hornblende content, the total proportion of heavy minerals (including epidote, rutile and titanite) remains stable from the Late Miocene to the Late Pleistocene and displays no significant variation.

**Figure 1:**
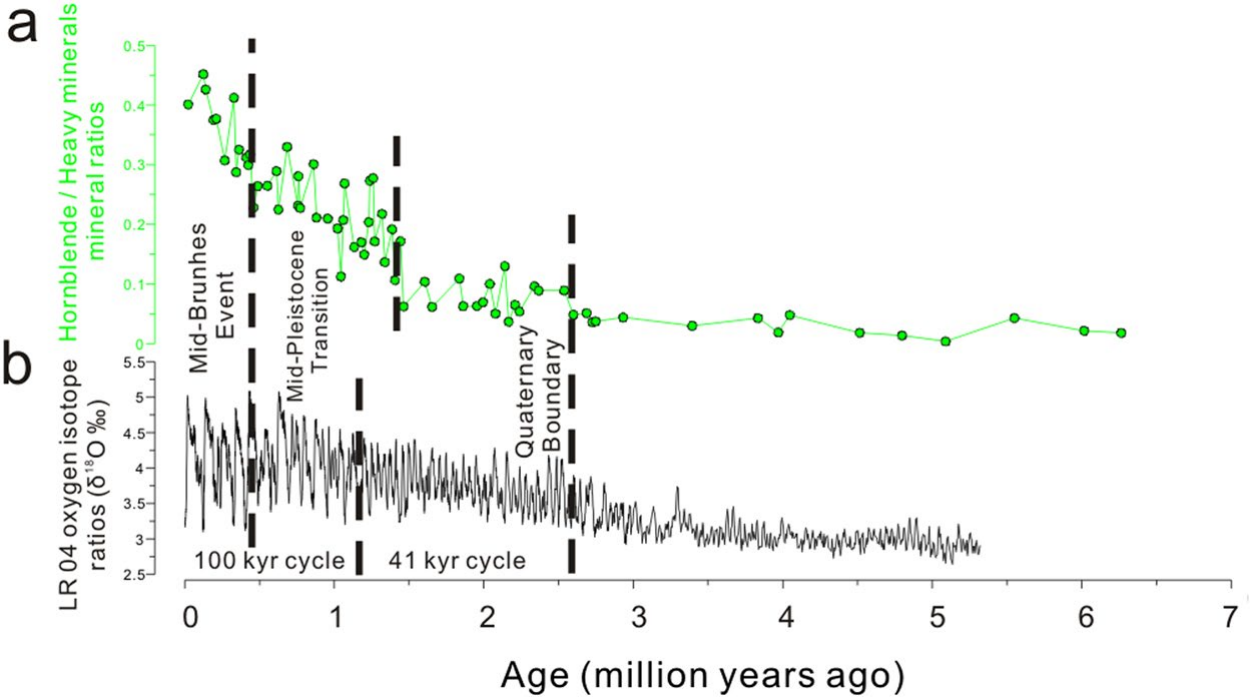
Comparison between **(a)** the R_h_ parameter and (**b**) the benthic δ^18^O record^62^. Two major climate transitions are marked with black dashed lines, including the onset of the Northern Hemisphere glaciation (2.6 Ma) and the Mid-Brunhes Event (0.5 Ma). A significant increase is observed at approximately 1.4 Ma, 0.2 Ma earlier than the mid-Pleistocene Transition (1.2 Ma) climatic boundary.

Within the long-term increasing R_h_ value trend, three boundaries can be recognized (Fig. 1 and Fig. S5). The R_h_ value remains low during the Late Miocene; the average R_h_ value for these samples is 0.03, and none of the samples exceeds a value of 0.05. A significant increase occurs at the Pliocene–Pleistocene boundary (approximately 2.6 Ma), with the average R_h_ value increasing to 0.09 and remaining at that value until 1.4 Ma. At 1.4 Ma, the R_h_ value increases by a factor of approximately 2–3 times that of the Pliocene baseline value, continuously increasing from a value of 0.18 at 1.4 Ma to a value of 0.47 at the end of the Pleistocene glaciation. Finally, a third increase is recorded in loess layer L5 (approximately 0.5 Ma) and is characterized by an almost threefold increase estimated using linear regression (Fig. S5).

The changes in the R_h_ record mimic the changes in global ice volume, as indicated by marine oxygen isotope records (Fig. 1). The first boundary, at 2.6 Ma, is generally accepted as the onset of the NHG. The 1.4 Ma boundary is close to the MPT at 1.2 Ma, when the glacial cycles transitioned from symmetric 41-kyr oscillations to strongly asymmetric 100-kyr oscillations^28^. However, the R_h_ record precedes the MPT boundary by approximately 0.2 Ma. The final boundary correlates well with the Mid-Brunhes Event (MBE) (0.5 Ma), which signals the amplification of glacial–interglacial oscillations.

## Discussion

### Use of R_h_ as a proxy for physical/chemical weathering

**Figure 2:**
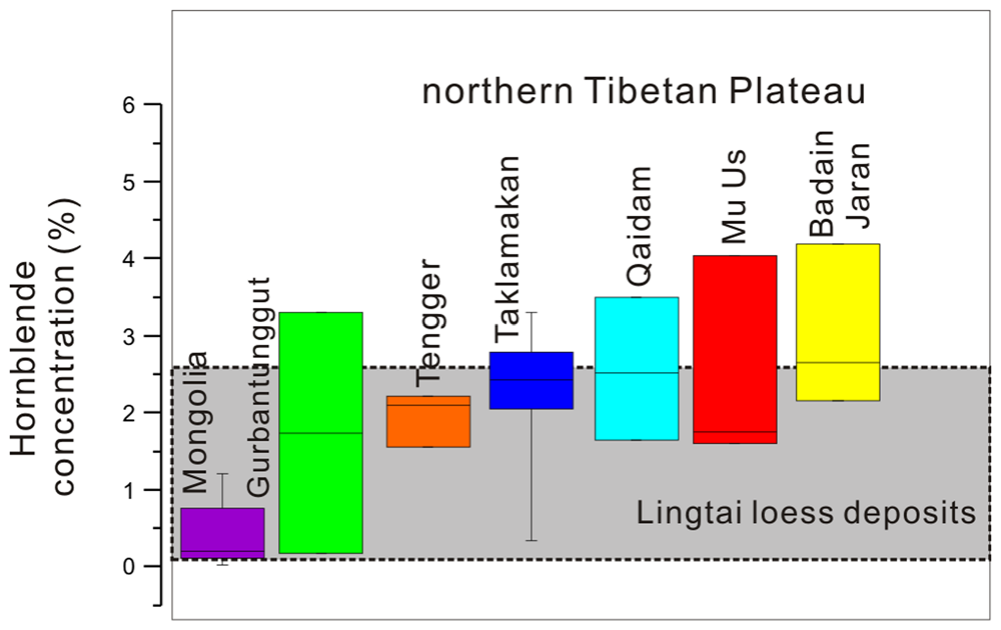
Distribution of the hornblende concentration in dust source deserts of East Asia. Hornblende concentrations are shown for each individual desert. The boxes represent the two inner quartiles of the data, and the whiskers extend to the minimum and maximum data points. The median of the data is indicated with a horizontal line inside the box. The hornblende concentrations are statistically distinguishable between these different sites. The data from the Taklamakan, Qaidam, Badain Jaran, Tengger and Mu Us deserts are from the northern Tibetan Plateau. These sites yielded significantly higher hornblende content than the Mongolian Gobi Desert. The data range for the Lingtai section is shaded.

Our analysis of desert sediments reveals spatial variations in hornblende abundance (Fig. 2). Five deserts located near the newly uplifted northern edge of the northern Tibetan Plateau, including the Badain Jaran, Tengger, Mu Us, Qaidam and Taklamakan deserts, have the highest content of hornblende minerals, ranging from 0.34% to 4.18%. A simple statistical analysis suggests that nearly all of the samples from these five deserts have a hornblende content greater than 1.5%. The hornblende content in the Gurbantunggut Desert varies widely and is not discussed here because the Gurbantunggut Desert is an area not prone to frequent dust storms^29^. In contrast, the abundance of hornblende in samples from the Mongolian Gobi Desert is relatively low, with five of the samples recording abundances lower than 0.8% and the final sample recording a maximum abundance of 1.2%. The low hornblende content of sands in the Mongolian Gobi Desert can be explained by the different tectonic setting of this desert compared to the northern edge of the northern Tibetan Plateau.

**Figure 3:**
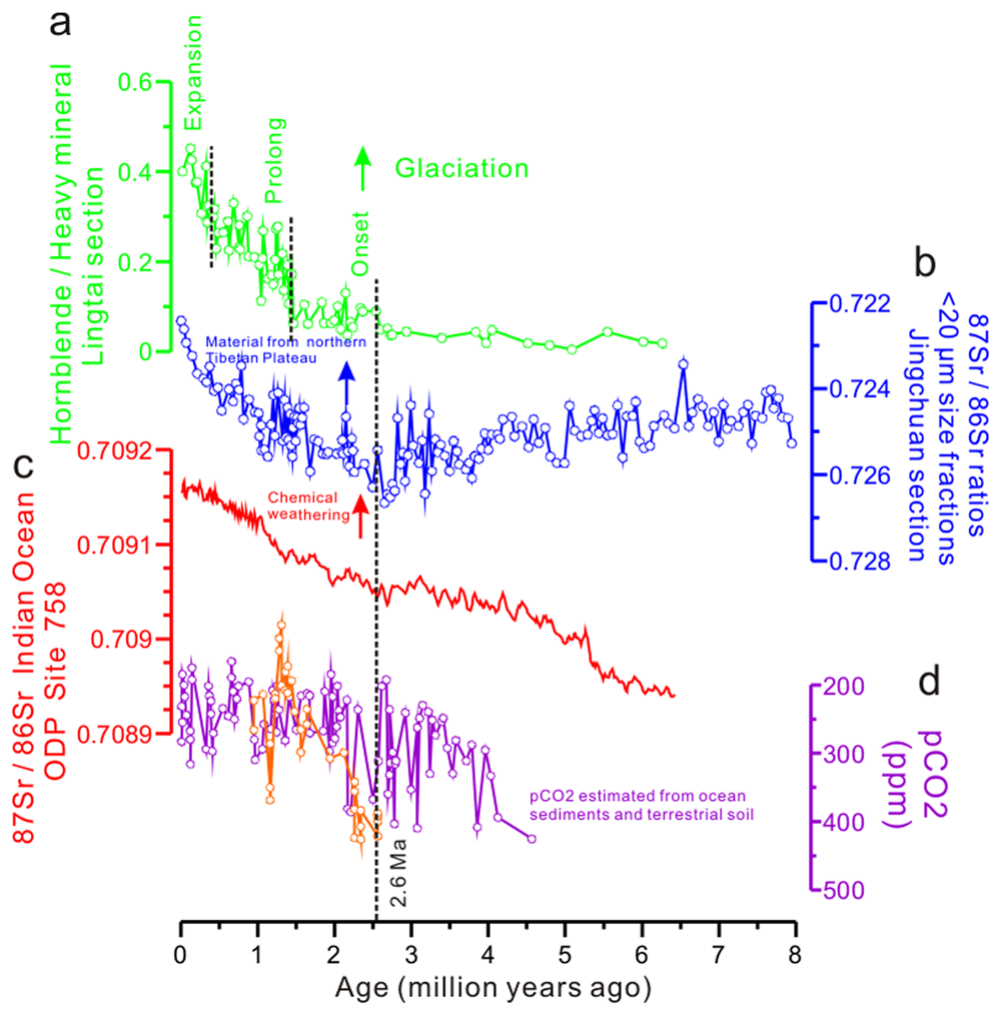
Comparison of the R_h_ proxy records from loess deposits in the Lingtai section to different parameters. (**a**) R_h_ proxy records for the past seven million years. (**b)** ^87^Sr/^86^Sr isotopic ratios in the Jingchuan section^37^, which reflect the contribution of materials from the northern Tibetan Plateau. ^87^Sr/^86^Sr values for the loess are plotted in light blue for reference. Materials from a relatively young orogen can result in lower ^87^Sr/^86^Sr ratios in sediments. **(c)** ^87^Sr/^86^Sr ratios in marine sediments from the Indian Ocean^57^; these values are much lower than those in materials from the Tibetan Plateau^58^, but they begin to increase after the Pliocene–Pleistocene boundary. **(d)** Atmospheric CO_2_ levels estimated from marine sediments, shown in purple^60^; CO_2_ levels estimated from terrestrial soil carbonate indices, shown in orange^61^. Atmospheric CO_2_ levels show a large decrease after 2.6 Ma.

Tectonic evidence in Mongolia suggests that the most recent deformation in this area has occurred since the Late Miocene as a result of the far-field effects of the Cenozoic India–Asia collision nearly 2000 km to the south^30, 31^. Thermochronology and field data further constrained the current uplift of the Gobi Altay and Altay mountain ranges in Mongolia, which began 5 ± 3 Ma^32^. The regional tectonic deformations promoted mountain denudation, thus enhancing the contribution of eroded materials to the basin. However, the Gobi Altay and Altay flat-topped massifs are tectonically unique. Unlike contractional or extensional orogens, there is no orogenic foreland or hinterland^33^, and the erosion rate is very low in this region^34^. Due to the very low erosion rate in the Mongolian Altay Mountains, the sediments in the Mongolian Gobi Desert thus have low hornblende content (Fig. 2). The high mountains along the northern Tibetan Plateau (e.g., the Kunlun Mountains and Qilian Mountains) are separated by foreland basins filled with kilometre-thick Tertiary and Quaternary sediments, implying very high erosion rates since that period^16^. We are thus able to use the spatial distribution of hornblende minerals in the loess stratigraphy to trace the loess deposits back to their source regions. The increase in the hornblende content along the Lingtai profile (Supplementary Fig. 4S-a) suggests that the contribution of hornblende from the northern Tibetan Plateau has increased relative to that of the Mongolian Gobi Desert. This is supported by the ^87^Sr/^86^Sr isotope record at the nearby Jingchuan loess section. In principle, Ca-bearing plagioclase is enriched in non-radiogenic ^86^Sr^35^. The Ca-bearing plagioclase was periodically rejuvenated by the input of erosion products from newly uplifted mountains along the northern Tibetan Plateau during glacial periods^36^. As discussed by Sun *et al*.^37^, the ^87^Sr/^86^Sr ratios in loess deposits reflect the ratio of contributions to the loess from exposed bedrock at high relief to contributions from recycled sediments from desert basins. The strong agreement between the R_h_ proxy and ^87^Sr/^86^Sr records in the loess (Fig. 3a and b) could be explained by the selective nature of Sr-bearing minerals (for example, plagioclase and hornblende, which are enriched in non-radiogenic ^86^Sr^35^). However, this is not the case during the Late Miocene to Pliocene. The ^87^Sr/^86^Sr ratios gradually increase (Fig. 3), whereas the R_h_ proxy is essentially flat. This clear difference may be explained by limitations of the R_h_ proxy and ^87^Sr/^86^Sr records. Regarding the R_h_ proxy, greater chemical weathering or diagenesis in the Late Miocene–Pliocene red clay than in the Pleistocene loess–paleosol sequence reduced the hornblende concentration, and thus its function as a proxy for mountain erosion was weakened. In contrast, weathering of biotite, which is rich in radiogenic ^87^Sr, released ^87^Sr from the biotite minerals, which was absorbed by clay minerals^38^. This weathering may result in a slightly increasing trend in ^87^Sr/^86^Sr ratios from the Late Miocene to Pliocene.

In recycled sediments, hornblende is rare because hornblende crystals completely decompose within ten thousand years^39^. Easily weathered minerals derived from uplifted passive margin sequences, known as fresh minerals, enhance further breakdown of detrital grains, which then become available for chemical weathering^6^. Rapid mechanical erosion, in turn, increases the fraction of fresh minerals supplied to the weathering zone, which will further increase the weathering rate^40^. Therefore, changes in the hornblende content along the Lingtai profile could reflect changes in the mountain erosional processes along the northern Tibetan Plateau.

The change in the hornblende content over time could also reflect the effects of other factors, including wind sorting, chemical weathering in the desert basin, and diagenetic alteration after loess deposition. The use of the R_h_ proxy allows us to minimize the signals from other factors to examine the effects of a single process. First, we can minimize the effect of wind sorting from our results due to the similar densities and shapes of hornblende and the heavy minerals; this negates the possibility that dust particles were lifted by different velocities of wind in the desert basins or that a variation in wind strength variably affected minerals of different densities and shapes during dust transportation. In addition, the samples were all treated by sieving (Methods Summary), and the heavy minerals, e.g., hornblende, epidote, rutile and titanite, were constrained within a narrow particle size range. This treatment should minimize the influence of particle size on the R_h_ value. Second, hornblende crystals are less resistant to chemical weathering than are other heavy mineral species and thus possess a shorter exposure time. The use of the R_h_ proxy therefore allows us to partially isolate contributions from crystalline bedrock in mountain belts mixed with recycled sediments, thereby enabling us to more accurately track changes in mountain erosion rates than could be accomplished using hornblende alone. Third, although the total proportion of heavy minerals (including epidote, rutile and titanite) remains stable in the long-term trend, the R_h_ proxy shows the glacial–interglacial cycles more clearly than using hornblende alone (Supplementary Fig. 4S-b).

Recent mineralogical studies on the Loess Plateau^41, 42^ suggest that the hornblende content at the northern Xifeng profile site is significantly higher than that at the southern Chaona profile site, consistent with modern climate patterns. This finding suggests that hornblende content is controlled more by chemical weathering and/or diagenesis rather than dominantly by erosion. Furthermore, a study by Nie and Peng (2014) indicates that the hornblende minerals in the late Quaternary loess deposits were not weathered; in contrast, they were strongly weathered in the early Quaternary deposits^43^. This suggests that the gradually increasing trend in the R_h_ record may reflect time-accumulated diagenesis. In general, chemical weathering during the diagenetic history produces a trend of smoothly increasing minerals migrating upwards through a sedimentary stratigraphy^44^, which could not account for the three jumps in the R_h_ record, in particular the doubling in value after 1.4 Ma. Unfortunately, the diagenetic effect could not be removed from the R_h_ proxy record due to the limitations in our present data. Therefore, we focus on the three boundaries in the R_h_ record to discuss their possible forcing mechanisms.

### Possible mechanisms for the three increases in the R_h_ record

As shown in Fig. 3a, changes in hornblende mineral distributions in the Lingtai section were closely associated with the onset, prolongation and expansion of the Northern Hemisphere ice sheets. Previous research has demonstrated the existence of two significant increases in mountain erosion, occurring at the onset of the NHG^4^ and the MPT^7, 8^; here, we present evidence for another increase occurring at the MBE if the R_h_ record can be regarded as a proxy for mountain erosion. The close relationship between the R_h_ record and climate change is further supported by an additional line of evidence that the R_h_ proxy record also mirrors glacial–interglacial cycles (Supplementary Fig. 4S-b). Glacial periods correspond to higher R_h_ values, and vice versa, suggesting that more easily weathered minerals are produced during periods of advanced mountain glaciation.

Our results demonstrate that there is a connection between the mountain erosion and climate records, but the reasons for this are unclear. We propose several hypotheses to explain this connection. First, we hypothesize that global climate cooling was strongly associated with the development of mountain glaciers at a regional scale, particularly in the high mountain belts around the northern Tibetan Plateau. This hypothesis may explain why the R_h_ proxy and marine isotope records both display similar long-term trends and glacial–interglacial cycles. Second, we hypothesize that the major climate transitions in the loess dust source area, including the transition from 41-kyr to 100-kyr glacial cycles at the MPT, prolonged the glaciation duration, which allowed larger and longer-lived mountain glaciers to develop. The larger amplitudes of glacial–interglacial cycles after the MBE, as well as the development of larger mountain glaciers, could have enhanced erosion in mountainous regions. This heightened erosion may explain the increased amounts of easily weathered minerals we observe at these climate boundaries, although it is possible that mountain glacier-induced erosion rates may respond nonlinearly to the expansion of Northern Hemisphere ice sheets.

The mountain erosion on the northern Tibetan Plateau was also related to the late Cenozoic mountain belt uplift history, in addition to climate changes. Uplift of this region began approximately in the Eocene^45–47^, with further significant uplift believed to have occurred in the Late Miocene. Deposition in the Linxia Basin, which is a foreland basin, began at approximately 29 Ma and continued uninterrupted until 1.7 Ma^48^, indicating a long-term tectonic uplift and deformation process in this region. Since approximately 8 Ma, the sedimentary sequence recorded a rapid uplift, which caused stronger mountain denudation and sediment overfilling in the Linxia Basin^49^. Rapid uplift events at 3.2 Ma^50^ and 3.6 Ma^51^ were followed stepwise by episodic accelerated growths at 2.6 Ma, 1.8–1.7 Ma, 1.2–0.6 Ma and 0.15 Ma, based on magnetostratigraphic analysis^52^. The tectonic events at 2.6 Ma, 1.8–1.7 Ma and 1.2–0.6 Ma, coinciding with the long-term but stepwise global cooling, may also explain the changes in mountain erosion rate we observe here. A strong tectonic control on the erosion rates is further supported by sedimentological evidence. Since 2.6 Ma, the sedimentation of dust materials onto the Loess Plateau has shifted towards a higher dust deposition rate and coarser grain size^53^. A sedimentation pulse characterized by sharp increases in the dust deposition rate and grain size also occurred at approximately 1.0 Ma throughout the Loess Plateau^54^. These evident changes in the dust deposition rate and grain size largely support an increase in silt supply and production in the source area. However, the R_h_ record does not capture all of the possible uplift events in the northern Tibetan Plateau, e.g., the event at 0.15 Ma^52^, which is likely due to two reasons: (i) the relatively lower resolution of the R_h_ record and (ii) other uplift events are not as strong as the three events presented here.

The Yellow River is one of the largest rivers in the world, and originates in the northern Tibetan Plateau. Incision of the upper reaches of the Yellow River and its tributaries occurs in the loess source mountains. Magnetostratigraphic chronology of the seven terraces of the Yellow River indicates that the oldest terrace formed at approximately 1.7 Ma^55^. A strong geomorphic instability persisted from at least 1.7 Ma^41^. As a result, the incision of the river channels in the source mountains and in the Loess Plateau could have produced additional silt-sized sediments that were transported via the river and deposited in the nearby Mu Us Desert^56^. The enhanced transport of materials eroded from the upper river reaches along the northern Tibetan Plateau and may have increased the transport of easily weathered minerals, such as hornblende. This may potentially explain why the erosion rate doubled after 1.4 Ma.

The R_h_ proxy record appears to correlate with an improved chemical weathering record from ^87^Sr/^86^Sr in the Indian Ocean^57^ at the Pliocene–Pleistocene boundary, which has been used to infer greater global tectonism and the onset of Northern Hemisphere ice sheets at 2.6 Ma (Fig. 3c). The Tibetan Plateau has higher ^87^Sr/^86^Sr values (0.719–0.825)^58^ than the global sea water value (0.708–0.709)^57^, suggesting that the large step-like increase observed in the seawater ^87^Sr/^86^Sr curve could be explained by an increased erosion rate of Tibetan Plateau materials. However, there is a noticeable decoupling between the R_h_ proxy and the seawater ^87^Sr/^86^Sr records for the older part of the studied time series (the Red Earth Formation). It has been suggested that the provenance of the Red Earth Formation is quite different from that of the Quaternary loess deposits^37^. If that is the case, the R_h_ proxy, as an indicator for changes in mountain erosion rates on the northern Tibetan Plateau, would be insufficient to indicate materials eroded from mountains along the northern Tibetan Plateau for the Red Earth Formation.

Our work better constrains the interaction between tectonic uplift, climate change and mountain erosion. As high levels of physical erosion transport fresh mineral surfaces to the weathering environment, physical erosion is coupled with chemical weathering, which uses atmospheric CO_2_ to transform silicate minerals into oceanic carbonate sediments^59^. For example, at the onset of the NHG, the enhanced chemical weathering rate caused atmospheric CO_2_ levels to decrease by 100–200 ppm^60, 61^ (Fig. 3d). This supports the idea that a positive feedback loop exists between ice sheets, atmospheric CO_2_, and erosion of newly uplifted and exhumed materials. Given the high surface area of the fine-grained dust materials in the loess source region, significant increases in our proxy record at the three major climatic boundaries may indicate increased CO_2_ sequestration and reduced atmospheric CO_2_ levels. In this sense, the increase in erosion played a key role in these three major climate transitions.

## Electronic supplementary material

Supplementary Information

Dataset 1D
