# Supplementary material for: A seven-million-year hornblende mineral record from the central Chinese Loess Plateau
Source: Sci Rep. 2017 May 24;7:2382. doi: 10.1038/s41598-017-02400-0 (PMC5443836; doi:10.1038/s41598-017-02400-0)
Supplement: Supplementary file 1 — Supplementary Information [file 41598_2017_2400_MOESM1_ESM.pdf]

Supplementary Information

A seven-million-year hornblende mineral record from the central Chinese Loess Plateau

Tong He*, Lianwen Liu, Yang Chen, Xuefen Sheng, and Junfeng Ji

Key Laboratory of Surficial Geochemistry, Ministry of Education, School of Earth Sciences and Engineering, Nanjing University, Nanjing, 210026, China.

Correspondence: hetong@nju.edu.cn

Methods Summary

Each sample was pretreated by adding 0.5 mol/L HCl and washing in an ultra-sonic bath for 30 minutes to remove carbonate cement and clay minerals. After sieving, a portion of the 28-75 μm size fraction particles were first placed in a round mold, to which was then added 20 ml of resin and 5 ml of hardener. After curing overnight, the round resin block was polished, carbon-coated and measured with a Mineral Liberation Analyzer (MLA). We used a MLA 650 system (composed of a Quanta 650 SEM with dual EDAX EDS detectors). The MLA measurement type was set to XBSE at a magnification of 800 and a frame resolution of 1024 x 800 pixels. The maximum number of particle sections to be measured was set to 40,000. Particle sections containing less than 100 pixels (equivalent to an 11.2 μm diameter particle section) were rejected to reduce the bias in calculated association data. The EDS parameters were set to collect a minimum of 2000 counts at count rate of 90000 CPS. All major minerals in the sample were identified, and their standard X-ray spectra were collected to be used after measurement data processing. In the final results, the total "unknown" minerals were <0.1% of the sample. We removed all identified clay aggregates that survived the acid leaching. The raw output of MLA provided the area percent for each mineral, which was converted to weight percent using known mineral densities. The "MLA processing tool" summed the pixel area for each mineral, reported it in square microns, and multiplied the summed area by a known mineral density to obtain the weight percent of each mineral in the sample.

Sampling resolution in the Lingtai section

To make our data set more representative of the section, we obtained duplicate samples from layers L2, L4, S4, L5, L7, S14 and L15. We also measured three samples from the S5 subdivided

layers (S5-1, S5LL1 and S5-3). The other 40 samples were measured from corresponding loess/paleosol layers. Fifty-seven samples from the Quaternary loess deposits (2.6-0 Ma) were analyzed. This sampling density is high enough to clarify long-term trends and to resolve glacial-interglacial variations in fresh mineral contents. Fifteen samples were obtained from the underlying Pliocene Red Clay sequence; this provides us with a low-resolution data set that is nevertheless capable of revealing general patterns.

Calculation method for anorthite percentages in plagioclase sub-species

We collected standard X-ray data for each plagioclase sub-species to obtain their elemental compositions; they are shown in the following table and were calculated using CaO percentages of 3.77% in oligoclase, 7.16% in andesine, 9.3% in labradorite and 16.4% in bytownite. The anorthite components in plagioclase are calculated as:

anorthite percentages in plagioclase = (3.77%*oligoclase%+7.16%*andesine%+
9.3%*labradorite%+16.4%*bytownite%)/20.1%/plagioclase%.

* 20.1% is the theoretical percentage of CaO in pure anorthite.

Table Plagioclase sub-species and their elemental compositions for loess samples of the Lingtai section.

	albite (n=11)	oligoclase (n=7)	andesine (n=7)	labradorite (n=4)	bytownite (n=2)
Na_2O	10.36	8.69	6.67	5.36	1.07
Al_2O_3	21.08	24.38	27.75	29.91	37.05
SiO_2	68.11	63.08	58.42	55.41	45.51
CaO	0.45	3.77	7.16	9.32	16.38
Total	100.00	100.00	100.00	100.00	100.00

Description of Dataset 1D

A separate "xls" file, Dataset 1D, was used to display the mineral contents for 23 mineral species: quartz, plagioclase (presented as the sum of the sub-species of albite, oligoclase, andesine, labradorite and bytownite), K-feldspar, micas and chlorite are most abundant. Accessory minerals include hornblende, epidote, rutile, titanite, schorl, zoisite, garnet, zircon and apatite. Oxide minerals, such as magnetite, Ti-magnetite, ilmenite and iron oxide, are listed separately. Minor pyrite particles were also detected. Additional minerals are grouped into "other minerals".

Fig. 1S Map depicting the potential loess source mountains, including Kunlun Mountain and Qilian Mountain (modified from Maher et al., 2009: Figure 1; republished with permission of [Geological Society of America], from [Magnetic and geochemical characteristics of Gobi Desert surface sediments: Implications for provenance of the Chinese Loess Plateau, Barbara A. Maher, 37(3), 2009]; permission conveyed through Copyright Clearance Center, Inc). These mountains have been subjected to the progressive surface uplift of the Northern Tibetan Plateau throughout the past few million years. The foreland desert basins for these mountains include the Taklamakan (TK), Qaidam (Qdm), Badain Jaran (BJ), Tengger (TGL) and Mu Us; these five desert basins are filled with freshly eroded materials from exposed bedrock in mountains. The Gurbantunggut (Gt) and Mongolian Gobi (MG) deserts are located in the north. The Chinese Loess Plateau is located downwind from these foreland basins and receives their eolian deposits. Lingtai and Jingchuan loess sections, as well as the 30 sampling sites from the seven deserts, are pinpointed with solid black squares.

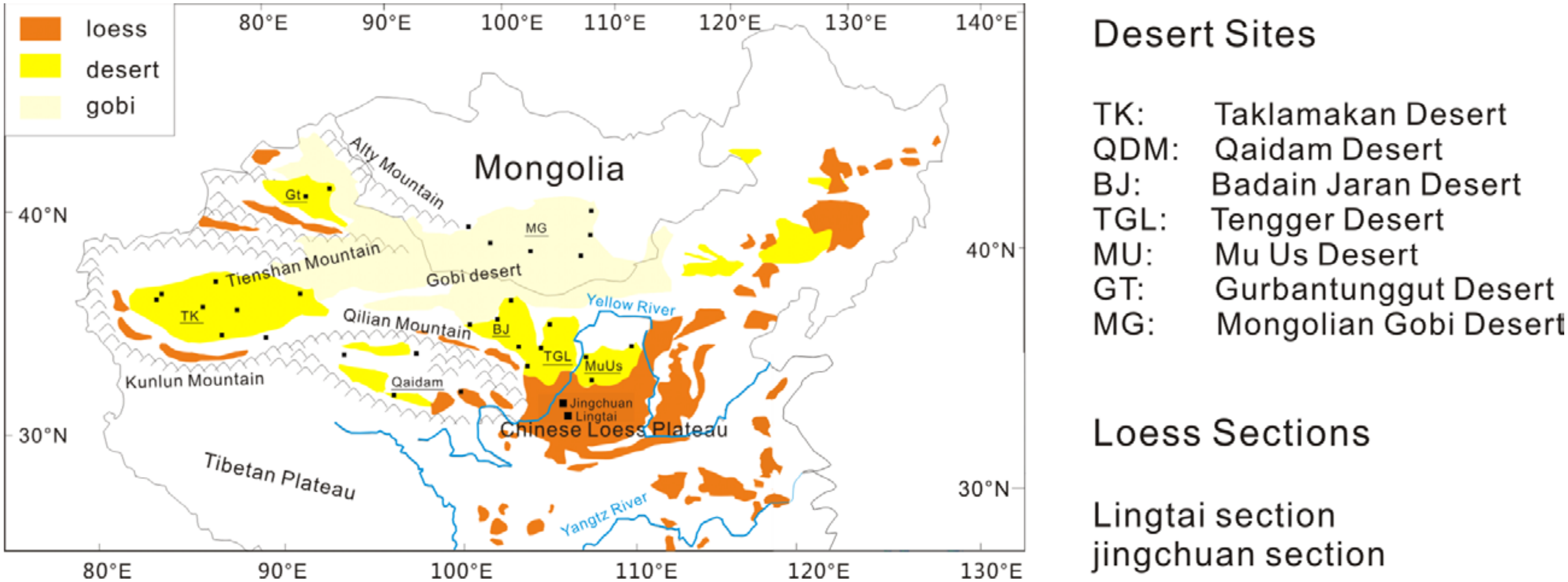
loess
desert
gobi
80°E
90°E
100°E
110°E
120°E
130°E
140°E
40°N
30°N
Mongolia
Alty Mountain
Tienshan Mountain
Gobi desert
Qilian Mountain
Kunlun Mountain
Tibetan Plateau
Yellow River
Yangtz River
Chinese Loess Plateau
Jingchuan
Lingtai
Gt
MG
TK
BJ
TGL
MuUs
Qaidam
Desert Sites
TK: Taklamakan Desert
QDM: Qaidam Desert
BJ: Badain Jaran Desert
TGL: Tengger Desert
MU: Mu Us Desert
GT: Gurbantunggut Desert
MG: Mongolian Gobi Desert
Loess Sections
Lingtai section
jingchuan section

Fig. 2S Stratigraphy of the upper Quaternary loess/paleosol deposits (left) and the lower Pliocene Red Clay formation (right). Magnetic susceptibility (MS) variation indicates the loess/paleosol cycles. Samples were selected according to their lithology, and their locations are noted in the MS curve as red solid circles. Loess layers (expressed as Li, where i=1, 2, 3, …, 33) are separated by paleosol layers (expressed by Si, where i=0, 1, 2, …, 32). The Bruhnes-Matuyama reversal occurs at the base of the L8 layer, and the Jaramillo subchron lies in the upper part of layer L10. The Olduvai subchron is constrained in layer L25, and the Matuyama/Gauss magnetic reversal occurs in loess unit L33 (Ding et al., 1999).

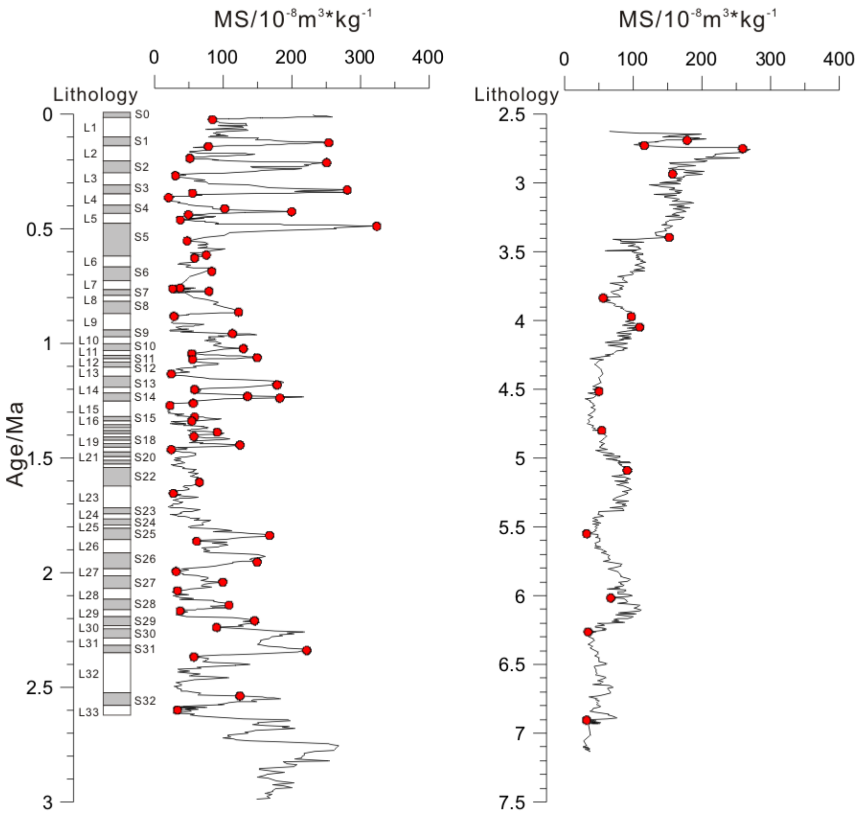

Fig. 3S Classification of mineral species by MLA. Sample LT 1852 is used here as an example; in total, 39104 particles were counted for this sample. These particles are shown here, along with their quantities: **(a)** Quartz, 19723; **(b)** Hornblende, 661; **(c)** Albite 6802; and **(d)** Bytownite, 52. Using MLA, we identified a total of 23 mineral species in the dust samples.

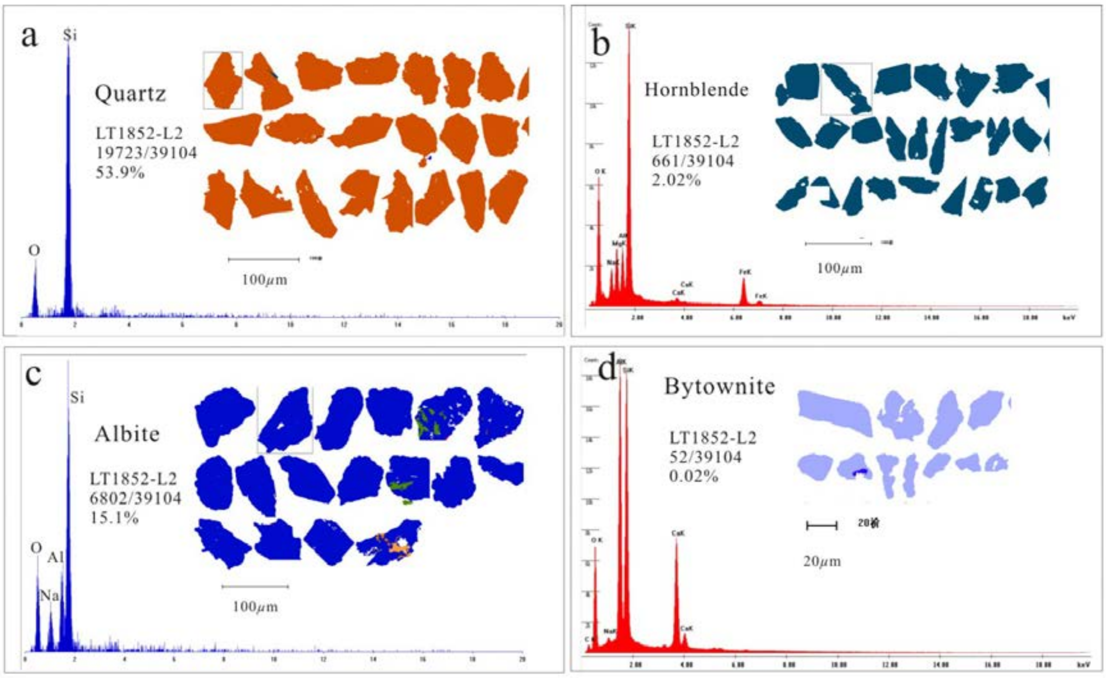

Fig. 4S Long-term changes in composition of easily weathered minerals over the past seven million years. **(a)** Easily weathered mineral compositions are expressed as plagioclase *versus* quartz, anorthite percentages in plagioclase, and total hornblende content. In Quaternary loess, the geochemical parameters begin to exhibit a long-term increasing trend. **(b)** Variability in alternations between glacial/interglacial cycles is shown in plagioclase *versus* quartz, anorthite percentages in plagioclase, and hornblende *versus* heavy minerals. These variations are less significant than they are in the benthic $\delta^{18}O$ record, which shows larger amplitudes in the glacial/interglacial variations.

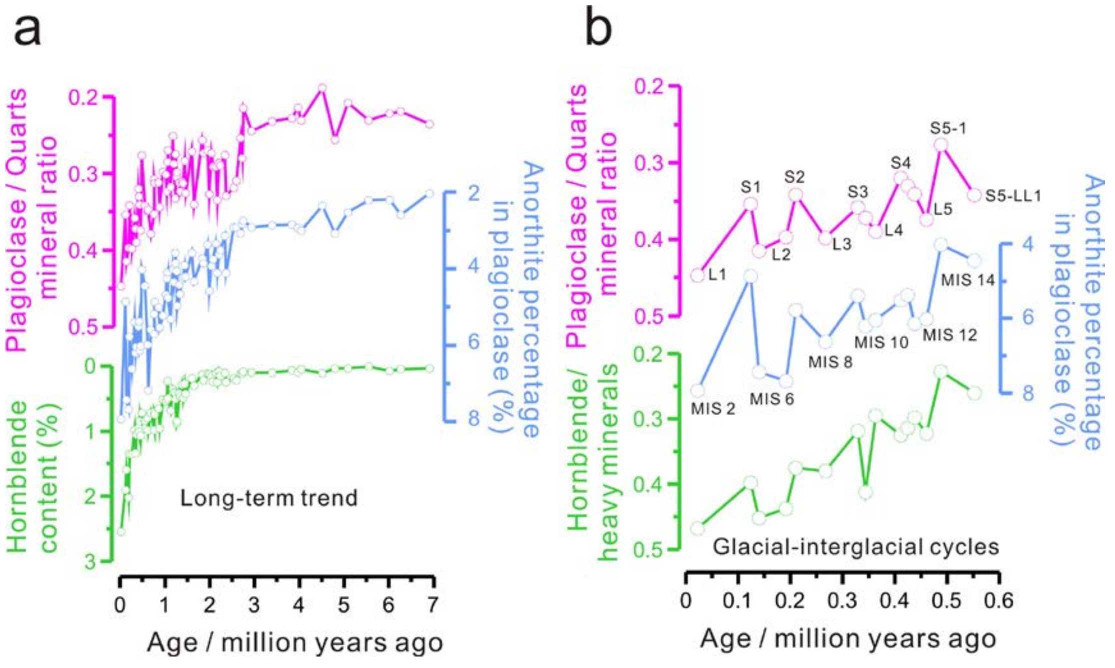

Fig. 5S Hornblende contents *versus* heavy minerals over the past seven million years. The three boundaries, shown as the transition points between lines of different colors, were determined by linear regression analyses. After ~0.5 Ma, erosion rates increase nearly three-fold from their pre-0.5 Ma values (from 0.12 myr^{-1} up to 0.37 myr^{-1}).

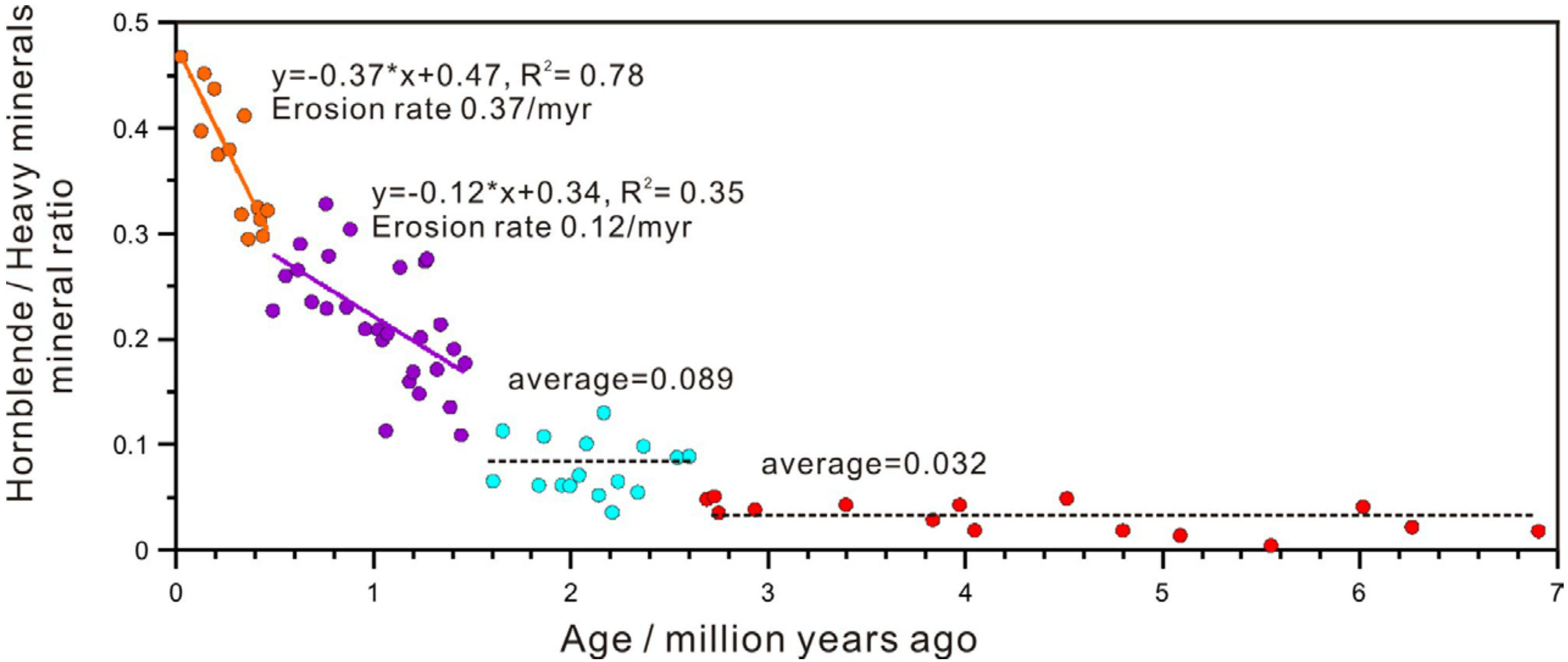

References

Maher, B. A., Mutch, T. J. & Cunningham, D. Magnetic and geochemical characteristics of Gobi Desert surface sediments: Implications for provenance of the Chinese Loess Plateau. *Geology* **37**, 279, doi:10.1130/G25293A.1 (2009).

Ding, Z. L. *et al.* Pedostratigraphy and paleomagnetism of a ~7.0 Ma eolian loess–red clay sequence at Lingtai, Loess Plateau, north-central China and the implications for paleomonsoon evolution. *Palaeogeography, Palaeoclimatology, Palaeoecology* **152**, 49-66, doi:10.1016/s0031-0182(99)00034-6 (1999).
